# How is leadership behavior associated with organization-related variables? Translation and psychometric evaluation of the implementation leadership scale in German primary healthcare

**DOI:** 10.1186/s12913-022-08434-z

**Published:** 2022-08-20

**Authors:** Sara Söling, Holger Pfaff, Ute Karbach, Lena Ansmann, Juliane Köberlein-Neu, AdAM-Study Group, Petra Kellermann-Mühlhoff, Lara Düvel, Till Beckmann, Reinhard Hammerschmidt, Julia Jachmich, Eva Leicher, Benjamin Brandt, Johanna Richard, Frank Meyer, Mathias Flume, Thomas Müller, Ferdinand M. Gerlach, Christiane Muth, Ana Isabel Gonzalez-Gonzalez, Kiran Chapidi, Robin Brünn, Peter Ihle, Ingo Meyer, Nina Timmesfeld, Hans J. Trampisch, Renate Klaaßen-Mielke, Jale Basten, Wolfgang Greiner, Bastian Suhrmann, Alexandra Piotrowski, Karolina Beifuß, Sarah Meyer, Daniel Grandt, Simone Grandt

**Affiliations:** 1 Institute of Medical Sociology, Health Services Research, and Rehabilitation Science, University of Cologne, Faculty of Medicine and University Hospital Cologne, Faculty of Human Sciences, Eupenerstr. 129, 50933 Cologne, Germany; 2 Center for Health Economics and Health Services Research, Schumpeter School of Business and Economics, University of Wuppertal, Wuppertal, Germany; 3 Department of Sociology in Rehabilitation, Faculty of Rehabilitation Sciences, Technical University Dortmund, Dortmund, Germany; 4 Division for Organizational Health Services Research, Department of Health Services Research, School of Medicine and Health Sciences, University of Oldenburg, Oldenburg, Germany

**Keywords:** Digital technology, Leadership, Change management, Organizational culture, Innovation climate, Social Capital, Medication therapy management

## Abstract

**Background:**

The Implementation Leadership Scale (ILS) was developed to assess leadership behavior with regard to being proactive, knowledgeable, supportive, or perseverant in implementing evidence-based practices (EBPs). As part of a study on the implementation of a digitally supported polypharmacy management application in primary care, the original ILS was translated and validated for use in the German language.

**Rationale:**

This study aimed to translate the original ILS into German and evaluate its psychometric properties.

**Methods:**

The validation sample consisted of 198 primary care physicians in a cluster-randomized controlled trial in which the intervention group implemented a digitally supported clinical decision support system for polypharmacy management. The ILS was assessed using a 12-item scale. The study included a process evaluation with two evaluation waves between 2019 and 2021. The ILS was used within this process evaluation study to assess the leadership support with regard to the implementation of the polypharmacy management. The ILS was translated in a multi-step process, including pre-testing of the instrument and triple, back-and-forth translation of the instrument. We tested the reliability (Cronbach’s alpha) and validity (construct and criterion-related validity) of the scale.

**Results:**

The four-dimensional structure of the instrument was confirmed (comparative fit index = .97; root mean square error of approximation = .06). Convergent validity was demonstrated by correlations with organizational innovation climate, social capital, and workload, which was consistent with the proposed hypothesis. Criterion-related validity of the ILS was demonstrated by predicting the organizational readiness for change scores using structural equation modeling. The reliability of the scale was good (α = .875).

**Conclusion:**

The German version of the ILS created in this study is a reliable and valid measure. The original four-dimensional structure of the ILS was confirmed in a primary care setting. Further psychometric testing is needed to establish the validity and reliability of the ILS and to transfer it to other health care settings. It is a useful tool for identifying the areas for implementation leadership development. Further research is needed on how, why, and when distinct types of leadership behaviors have different effects on healthcare organizations in implementation processes.

## Background

Implementing change in healthcare organizations can be challenging. In recent decades, however, there has been a paradigm shift from a simplified and static understanding of implementation processes to a more complex and dynamic one. Hunter (2020) argued that successful implementation comprises the dynamic interplay of facilitating conditions, innovation, recipients, and context [1]. In this complex interplay of significant factors, implementation-supportive leadership behavior is important for a successful change process in healthcare organizations.

In addition to the theoretical assumptions, empirical evidence supports the significance of the role of leaders in the implementation process [2, 3]. Particularly, the full-range leadership model (FRLM), which includes a typology of leadership behaviors such as transformational, transactional, non-transactional laissez faire leadership, has often been used as a conceptual basis in research for investigating correlations between leadership and organizational performance [4–6]. Leadership influences multiple factors in the organizational context – such as culture, communication, networks, and resources – and is the key enabler in creating a climate conducive to the implementation of EBPs [7].

In implementation research, these relevant implementation factors can be mapped to a proven framework for investigating change processes – the consolidated framework for implementation research (CFIR). In the field of health services studies, the conceptual approach of CFIR has often been used to guide and facilitate, plan, and evaluate the implementation of evidence-based practices (EBPs) [8, 9]. From a meta-theoretical perspective on implementation research, this framework provides a compilation of constructs that have been associated with effective implementation [2]. Determinants are grouped within five main domains relevant for implementation research: the intervention, inner setting, outer setting, individuals involved, and process by which implementation is accomplished. The domain “inner setting” contains relevant constructs to capture the internal dynamics of the organization in a focused manner. One important element of the “inner setting” is leadership, which is also linked to the construct of “implementation climate” and “implementation readiness”. Leadership in particular and the theories associated with it play an important role in explaining the translation of theory into practice and have evolved and have been integrated into implementation research [10, 11].

In addition to a theoretical conceptualization of leadership in implementation research, there is a body of theoretical literature in health services research: the knowledge translation and exchange literature [12–15]. In this research area, change processes are examined primarily with regard to the exchange of knowledge during change. In the dynamic and interactive change process, the ability to transfer knowledge is defined as a core competency of the leader [13, 16]. Because the nature of knowledge transfer processes is associated with diverse boundaries, the leader needs to be able to recognize and use new knowledge (absorptive capacity). The degree of leaders’ absorptive capacity in knowledge transfer promotes or inhibits organizational learning and facilitates or prevents the successful implementation of change processes at the organizational level [17, 18].

### Implementation leadership: a new concept

Implementation leadership (IL) is a recently emerged concept developed on the basis of literature on organizational climate and cultural change [19, 20]. It has been operationalized in using different theoretical leadership models [21, 22]. The Implementation Leadership Scale (ILS), which is the focus of this paper, is based on the full-range leadership model, which includes aspects of the transformational leadership theory, as in the theoretical development of the scale indicated by Aarons et al. (2014) [23]. Other conceptual approaches related to IL include the behavioral leadership model and the Ottawa model of implementation leadership [24] or the hierarchical framework of leadership behaviors [25]. The models and related items differ particularly in their description of the leadership behaviors being measured.

The ILS measures leadership types in the context of EBP implementation. It has been used in many countries such as Greece, Norway, and China and in various settings (e.g., nursing or mental health care settings), and its validity was confirmed [26–28]. It focuses on leader behaviors related to organizational culture and climate-embedding mechanisms that promote strategic climates for EBP implementation. In addition, leadership behaviors that focus on a strategic imperative related to an implementation outcome such as adopting or applying an EBP may influence team members’ attitudes and behaviors regarding the imperative [23]. The four types of leadership behavior in ILS represent specific leadership behaviors – in contrast to general leadership behavior – that leaders may perform to facilitate EBP implementation, for example, removing obstacles to EBP implementation (proactive leadership); communicating benefits of EBP (knowledgeable leadership); recognizing, appreciating, and supporting employee efforts in learning and using EBPs (supportive leadership); and persisting through challenges in implementing EBP (perseverant leadership).

However, to our knowledge, no specific measurement tool is available for the primary care setting in Germany. As the purpose of the study was to identify a process evaluation measure that focused on our primary research question on evaluating barriers and facilitators in the implementation process, we sought a brief and specific measure related to the leadership behavior of primary care physicians (PCPs) – as a facilitating factor. To this end, we investigated the original ILS (leader version), which we translated, and evaluated its application in the German primary care context.

### Conceptual model

**Fig. 1:**
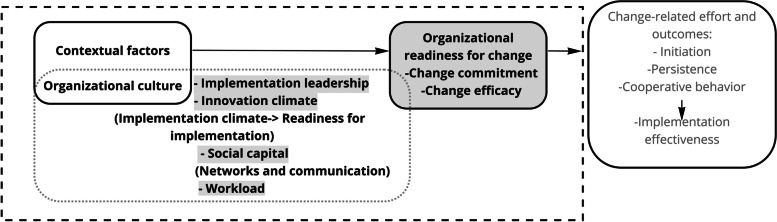
Conceptual Model. Notes: The subject matter of the present study = all measures colored in gray; determinants and outcomes of organizational readiness approach = all constructs in frames with solid lines; inner setting domain of CFIR (related constructs) = frame with dotted lines

Assumptions about the interrelationships among the constructs investigated in our study are mainly based on the CFIR and an organizational theory approach. As described by Weiner (2009) or Damschroder (2009), receptive organizational context factors or internal factors of organizations are determinants in the implementation process [29] (see Fig. 1).

Furthermore, two constructs related to the organizational level were investigated in our study. The items in these scales are formulated generically and do not directly address implementation activities (innovation climate, social capital). One scale is also described in the CFIR in a broader sense as a facilitating factor for implementation processes. It is assigned to the construct on “networks and communication processes” (social capital). In the context of the above classification and description of ILS and CFIR, the social capital scale represents a specific aspect of the networking and communication processes in an organization (CFIR). We hypothesize that social capital facilitates the implementation processes and may be positively associated with leadership [30]. In addition, we hypothesize that support for implementation through quality management implementation practices (measured by ILS) will foster a positive climate for innovation implementation (innovation climate) [19]. In a further step, we included variable workload in our model. It is not directly mentioned in the literature on which our theoretical–conceptual framework is based, but it is reasonable to assume that the workload of leaders increases. However, in our study, we assumed that there cannot be a positive relationship between the two factors. The variable used refers to workload related to general practice activities in primary care, which remained stable in our study (measured by the number of patient appointments during implementation). It therefore does not serve as a direct indicator of implementation activities.

The last construct that is examined in our study – implementation readiness – is operationalized through direct indicators of organizational commitment to the decision of implementing an innovation. These include leadership commitment at the micro level (measured by ILS) and organizational readiness at the meso level (measured by organizational readiness for implementing change [ORIC]). ORIC is conceptualized following Weiner (2009) and measures the extent to which members in an organization are psychologically and behaviorally prepared to implement organizational change [29, 31]. With reference to our study setting, we assume that the direction of the effect relationship runs from the micro (ILS) to the meso level (ORIC; predictive validity) [32]. Nevertheless, in some situations, leadership behavior may be independent of ORIC. Even if leaders initiate many implementation activities, ORIC may not necessarily be accomplished, for example, if appropriate resources are also not provided at the organizational level. In addition to resource allocation, temporal elements may also have a significant impact on the implementation process. Depending on the phase of the intervention (initiation phase versus implementation phase), the concentration of decision-making autonomy (centralization) by individual actors (e.g., leaders) was found to be negatively or positively associated with innovation [33, 34]. Consequently, the quality of micro- and meso-level relations over time will be decisive to the effectiveness of implementation.

The research questions arising from the conceptual model and setting of our study are as follows: 1) can the validity and reliability of the ILS be confirmed in primary care organizations? and 2) how is the ILS empirically related to social capital, innovation climate, ORIC, and workload in our sample of primary care organizations (construct validity, criterion-related validity)?

## Methods

### Study design and data collection

In this secondary analysis, data from two surveys were used to examine the psychometric parameters of the German version of the ILS. The data were collected as part of the formative evaluation accompanying the effectiveness study in the project “Application of a digitally supported pharmacotherapy management system” (AdAM project), which was conducted in PCP practices from 2017 to 2021 [35]. The design of the effectiveness study influenced the data collection of this study. Because the effectiveness study was a stepped-wedge, cluster-randomized controlled trial (cRCT) with open cohorts and the second survey exclusively addressed adopters, data of only a subset of the participating physicians in both surveys are available for longitudinal analyses.

Data for the ILS were collected in the first survey as part of an eight-page questionnaire, which included information on physicians’ attitudes regarding uptake of the intervention and other implementation factors. In addition to the ILS, it included a technology acceptance scale, demographic questions (e.g., gender, age, professional experience, and practice structure), and some questions that had been used in previous health services research studies. The results of these additional measurement tools were not used for validation, except for four measures: organizational innovation climate, social capital, workload, and ORIC. The second survey covered ORIC, process normalization, perceived implementation success, and practice resources. As data were documented pseudonymously, we were able to link both surveys at the participant level. All physicians with fully completed surveys on ILS, innovation climate, social capital, and workload measures at the first time point were included in the construct and criterion validity analyses. Only data sets from fully completed surveys on ILS and ORIC were included in the path analysis for testing predictive validity (data from the ILS at the first time point and from the ORIC at the second time point).

Participating physicians received the questionnaire by mail from the Association of Statutory Health Insurance Physicians of the Region Westphalia/Lippe. The inclusion criteria of this validation study were identical to the inclusion criteria of the formative evaluation study of the AdAM project. The data from the first survey were collected between November 2019 and January 2020. Data from the second survey were collected between September 2020 and December 2020. To increase the response rate, we used the tailored design approach by Dillman (1978), which means that physicians were reminded three times by e-mail to respond to the questionnaire [36]. The first questionnaire was pre-tested with PCPs in two stages: in think-aloud interviews (*n* = 4) to assess the comprehensibility of the questions and in a secondary sample survey by post (*n* = 10) to test whether the skip pattern had the desired effect and whether the entire range of the scales was used and not just one direction. On the basis of the results, minor modifications were made to the overall structure and presentation quality of the final questionnaire.

### Setting and sample characteristics

The setting examined in the study was that of outpatient care by PCPs, where a digitally supported and evidence-based clinical decision support system for polypharmacy management was implemented in the AdAM project. PCPs implemented evidenced-based practices—such as digitally supported clinical decision making and medication reviews—for patients in the intervention group at least once a year. The digital software application provides the possibility to update information (e.g., on new diagnoses and prescriptions not yet settled with the patient’s health insurance provider) and to add specific details that are not included in the data submitted to health insurance funds (e.g., height, weight, laboratory test results on renal function, over-the-counter drugs, and medication doses). PCPs then examined patients’ medication regimens, supported by alerts from the application in case of inappropriate prescriptions (e.g., drug–drug and drug–disease interactions, inappropriate dosages, or potentially inappropriate drugs because of the patient’s age).

**Fig. 2:**
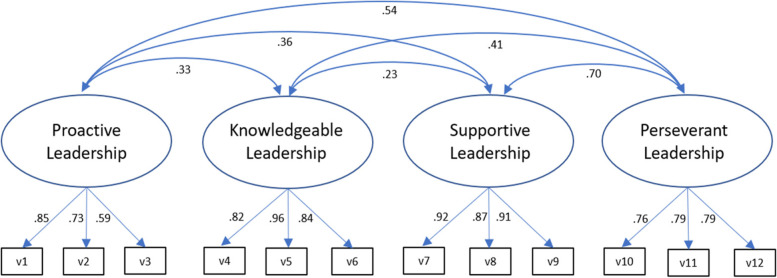
Factor loadings for the implementation leadership scale. Note: *n* = 198; all factor loadings are standardized and statistically significant, *p* < .001; χ^2^(48) = 84.59, *p* < .001; comparative fit index = .974; Tucker-Lewis index = .965; root mean square error of approximation = .062; standardized root mean quare residual = .051

The final sample size of the first and second surveys was 219 respondents (68.3% response rate) and 334 respondents (44.5% response rate), respectively. The final measurement model included 198 physicians (see Fig. 2) with complete data in the intervention group from 2018 to 2019 in the AdAM project. The path analysis model included 183 physicians at the first time point and 135 physicians at the second time point (see Table 3). For the second survey, it should be noted that the group was partly different from the first survey – owing to the study design of the effectiveness study (stepped-wedge cRCT with an open cohort). At the second measurement point, there were more participants in the intervention group who could be approached for the survey. But the lower response rate may be related to the fact that the second survey targeted more physicians who had just started using the software and could not yet provide ratings. Nearly all respondents of the first and second surveys were practice owners (92 and 94%, respectively) and were predominantly men (65 and 63%, respectively) and 50–60 years old (46 and 49%, respectively); the participants had an average of 17 years (survey 1) and 18 years (survey 2) of experience working as a PCP. The study population represents the potentially includable population of PCPs in the region where the intervention was implemented in terms of the distribution of sex and age. The distribution in terms of position within the practice (professional title) supports our intention to validate the leader version of the ILS, as almost all physicians were practice owners and therefore had a leadership role. Consequently, we considered this measurement tool as suitable to be validated with our sample.

### Translation of the implementation leadership scale

The translation process was guided by the recommendations of the World Health Organization for translating measures [37]. These recommendations call for a forward translation and then a back translation, supplemented by discussions on the translation process in which the terminology is discussed with regard to cultural differences in the meaning of the terms. Items were translated by bilingual translators from an independent translation agency. Every item of the scale was translated forward and back by three different bilingual translators. After the translation, three members of the research team rated the forward and back translations with regard to the comprehensibility of the translations for the German context. Each item was assessed and assigned a value of either 0 (no accordance), .5 (accordance, but not for all words), or 1 (accordance), which were summed up, and the final version was discussed. The translation of the scale was checked for comprehensibility in cognitive interviews with PCPs (*n* = 4) during the pre-test of the entire questionnaire. The respondents did not report any difficulties in understanding the individual items or technical terms relating to the ILS.

### Measures

#### Data aggregation

The instruments used have been developed to assess innovation climate, social capital, and organizational readiness at the organizational level of analysis. Physicians answered the organization-related aspects of our questionnaire as key persons of the participating practice. As recommended, measurement based on individuals’ assessments of collective capabilities is preferable when collective outcomes depend on skillful teamwork [38]. All organization-related instruments (organizational innovation climate, social capital, ORIC) and individual-related instruments (ILS, workload) had an adequate item structure (items were written from the perspective of the collective for organization-related instruments and from the perspective of the individual for individual-related instruments). For the above measurement tools, we did not aggregate data at the organizational level because almost all physicians in our sample were solo practice owners and did not work in group practices.

#### Implementation leadership scale

In the original English version, the four subscales are described following Aarons (2014): “proactive leadership” (items 1 to 3), “knowledgeable leadership” (items 4 to 6), “supportive leadership” (items 7 to 9), and “perseverant leadership” (items 10 to 12) [23]. The scores for each subscale were created by computing mean scores for each set of items related to a given leadership type, with higher scores indicating stronger leadership activities. To adapt the scale to a specific research context, items can be made specific by adding the name of the EBP. In our survey, the EBP was specified as the use of project software (including digitally supported evidence-based medication management). Physicians were asked to answer the Leader version of the ILS survey on a 5-point Likert scale from 1 (strongly disagree) to 5 (strongly agree; α = .875). The English items are presented in the Results section (α = .875; see Table 1).

**Table 1:** Characteristics of the implementation leadership scale, subscales, and item statistics

Item #	Mean (*SD*)	Factor loading (Std.)	Acceptance (Completion rate in %)	Corrected item-total correlation	Item difficulty
**Factor 1: Proactive leadership subscale**	2.23 (.96)				
1) I developed a plan to facilitate EBP implementation.	2.23 (1.17)	.845**	98.16	.596	44.6
2) I removed obstacles to implementation of EBP.	2.39 (1.16)	.731**	97.24	.534	47.8
3) I established clear department standards for implementation.	2.30 (1.15)	.592**	98.62	.576	46.0
**Factor 2: Knowledgeable leadership subscale**	3.54 (.92)				
4) I know about EBP.	3.62 (1.00)	.820**	98.16	.541	72.4
5) I am able to answer staff questions about EBP.	3.41 (1.02)	.959**	98.62	.554	68.2
6) I know what I am talking about when it comes to EBP.	3.44 (1.05)	.839**	98.16	.599	68.8
**Factor 3: Supportive leadership subscale**	3.34 (1.22)				
7) I recognize and appreciate employee efforts.	3.15 (1.38)	.916**	97.24	.739	63.3
8) I support employee efforts to learn more about EBP.	3.31 (1.28)	.871**	98.62	.763	66.2
9) I support employee efforts to use EBP.	3.42 (1.39)	.905**	96.78	.730	68.4
**Factor 4: Perseverant leadership subscale**	3.08 (.97)				
10) I persevere through the ups and downs of implementing EBP.	2.74 (1.21)	.758**	98.16	.719	54.8
11) I carry on through challenges of implementing EBP.	3.23 (1.16)	.790**	99.08	.691	64.6
12) I react to critical issues regarding implementation of EBP.	3.17 (1.06)	.790**	96.78	.728	63.4
**ILS Total**	3.07 (.74)				

Notes: *n* = 198, *SD* = standard deviation, ** *p* <.001; items were rated on a 5-point Likert scale ranging from 1 (strongly disagree) to 5 (strongly agree)

#### Organizational innovation climate

The “organizational innovation climate” scale consists of seven items and has been used in previous studies in health services research [39]. It measures the extent to which the organization’s current perceived climate promotes innovative ideas and behavior among employees. The items assess the extent to which the ideas, suggestions for improvement, and efforts of the employees regarding the introduction of an innovation are taken into account in the organization (PCP practice). Respondents had to choose one answer on a four-point Likert scale ranging from 1 (strongly disagree) to 4 (strongly agree; α = .804).

#### Social capital

The “social capital” scale consists of six items and has been recently validated as an employee version [30]. It has been characterized by different dimensions related to mutual understanding, warm circle, trust, “we-feeling” (i.e., a sense of being part of a team), mutual help, and shared values. The scale captures the enablement of a person to “coordinate their activities in an implicit and efficient way and to develop a healthy social climate.” The participants answered the items on a 4-point Likert scale ranging from 1 (strongly disagree) to 4 (strongly agree; α = .899).

#### Organizational readiness for implementing change

The “organizational readiness for implementing change” scale with its two subscales “change commitment” and “change efficacy,” proposed by Shea et al. (2014) [31], were recently translated and evaluated for the German context [40]. As recommended in the validation study of the German translation, we used a 9-item version of the scale instead of a 10-item version of the original scale. During the adaptation to the German context, the comprehensibility of item 10 remained low owing to inadequate translation of one term that has a strong cultural connotation and no equivalent phrase in German. Therefore, the item was omitted from the German version. The subscales capture the respondents’ perceived readiness for implementing change at an organizational level using five items (items 1 to 5 = change commitment; α = .967) and four items (items 6 to 9 = change efficacy; α = .956), respectively, each to be answered on a 5-point Likert scale ranging from 1 (strongly disagree) to 5 (strongly agree).

#### Workload

The item “workload” measured self-assessed perceptions of PCP workload over the past 2 weeks on a scale from 0 (not at all stressed) to 10 (very stressed; mean = 5.61, standard deviation = 0.20, confidence interval = 5.2–6.0). The item refers to the workload associated with general physician activities in primary care (e.g., diagnostic and therapeutic activities).

### Statistical analyses

To assess the psychometric quality of the ILS in primary care organizations, a two-step procedure was conducted [41]. In the first step, a confirmatory factor analysis (CFA) was used to examine whether the construct of implementation leadership in its four dimensions (namely “proactive leadership” [items 1 to 3], “knowledgeable leadership” [items 4 to 6], “supportive leadership” [items 7 to 9], and “perseverant leadership” [items 10 to 12]) can be confirmed by assessing global and local fit Indices. To test the prerequisites for factor analysis, the Kaiser–Meyer–Olkin (KMO) test for sampling adequacy and Bartlett’s test for sphericity were performed [41]. A factor analysis such as CFA is recommended to confirm content validity [29]. In the second step, bivariate analyses and structural equation modeling (SEM) were conducted to confirm criterion-related validity types such as convergent, discriminant, and predictive validity. Descriptive statistics were calculated for scale items (means, standard deviation, acceptance, inter-item correlations, corrected item-total correlations, item difficulty) and bivariate analyses (correlations; see Table 2). The maximum likelihood (ML) estimation procedure in Stata 15 software was used to test CFA and SEM, and Satorra–Bentler (SB) model fit measures were used to adjust more robust estimators for our study sample. No missing values were imputed for the final measurement model.

**Table 2:** Pearson product-moment correlations of Implementation Leadership Scale scores with organizational innovation climate and social capital scores (convergent validity) and workload scores (discriminant validity)

		Pro-active leadership	Knowledgeable leadership	Supportive leadership	Perseverant leadership	ILS total
Convergent validity	Organizational innovation climate	.311**	.262**	.300**	.479**	.435**
	Social capital	−.060	.082	.230**	.104	.156*
Discriminant validity	Workload	.005	−.040	.051	.020	.065

Note: *n* = 198, *p < .05, **p < .001

#### Content validity

The first step was the performance of a CFA with four factors for the entire data set. Factorial validity was verified by factor loadings of the 12 items of the ILS, where loadings ≤.71 were interpreted as excellent, ≤.63 as very good, ≤.55 as good, ≤.45 as fair, and ≤ .32 as poor [31]. The following thresholds were used to determine a good model fit: average variance extracted (AVE) ≥ .5, factor reliability ≥.6, reliability (Cronbach’s α) ≥ .7.

#### Criterion-related validity

It was not feasible in the present study to test convergent validity with a similar validated instrument. Therefore, we analyzed the relationships between the ILS and theoretically related measures in the “inner setting” domain (CFIR). Bivariate analyses of the ILS were examined through organizational innovation climate, social capital scores (convergent validity), and the workload measure (discriminant validity; see Table 2).

We conducted a path analysis using the ILS and its subscales and the ORIC and its subscales and testing the predictive validity of ILS. Predictive validity was defined as “the ability of a test to measure an event or outcome in the future” [42]. In the path analysis, the higher-order constructs and subconstructs were modeled as latent variables and linked to their associated measurable indicator variables. A series of global goodness-of-fit indices were used to assess the extent to which the observed data were explained by the proposed models: normed χ^2^-statistic (χ^2^/df ≤ 3), comparative fit index (CFI), and Tucker–Lewis index (TLI ≥ .95: acceptable; TLI ≥ .97: good), and root mean square error of approximation (RMSEA ≤.08: acceptable; RMSEA ≤.05: good).

## Results

### Content validity

Responses to the twelve items of the ILS ranged from 1 (strongly disagree) to 5 (strongly agree), with means ranging from 2.23 (item 1) to 3.62 (item 4) and standard deviations ranging between 0.75 and 1.39 (see Table 1). Most participants assigned a rating in the middle of the scale, with a slight tendency to agree. Internal consistency yielded a four-factor solution with a good Cronbach’s α = .875 (for factor 1: α = .774, for factor 2: α = .911, for factor 3: α = .918, for factor 4: α = .842), and inter-item correlation values ranged from .150 to .785 (for factor 1: *r* = .439 to −.635, for factor 2: *r* = .693 to .819, for factor 3: *r* = .762 to .816, for factor 4: *r* = .598 to .692). Overall corrected item-total correlations ranged from .534 to .763. Taking all items into account, more than 97% of the measures were answered. Item difficulty ranged from 44.6 (item 1) to 72.4 (item 4). Prerequisites for the factor analysis were met, as the comprehensibility of the translated scale was checked in cognitive interviews; KMO measure was .832, and Bartlett’s test of sphericity yielded a χ^2^ value 1447.20 (*p* < .001). This indicates that a factor analysis of the data was appropriate [30]. CFA for the hypothesized four-factor structure of the model demonstrated a good fit, as suggested by multiple goodness-of-fit indicators (*n* = 198; χ^2^(48) = 84.59, *p* < .001; CFI = .974; TLI = .965; RMSEA = .062; SRMR = .051). Factor reliability exceeded critical values (factor reliability = .77, .90, .92, and .82, respectively; AVE = .53, .76, .80, and .60, respectively).

CFA was used to reproduce the second-order CFA proposed by Aarons for the original ILS with our sample [23]. Because this analysis could not be performed with our sample, we accounted for the theoretically assumed relationships between the subscales by calculating the covariances and achieved a reasonable model fit (*n* = 198; *p* < .001; χ^2^(48) = 84.59, *p* < .001; CFI = .974; TLI = .965; RMSEA = .062; SRMR = .051). Figure 2 provides an overview of the standardized factor loadings and covariances between subscales for the four-factor model. Factor loadings ranged from .59 to .96, and all factor loadings were statistically significant (*p* < .001).

### Criterion-related validity

The results of the convergent validity analyses are summarized in Table 2. ILS total score and subscale scores were significantly correlated (*p* ≤ .001) with organizational innovation climate, with the correlations ranging from .26 to .48. Supportive leadership was the only ILS subscale that was significantly correlated with social capital scores (*p* ≤ .01; *r* = .23). All other ILS subscales individually had no significant correlations, whereas ILS total score was significantly correlated with social capital scores (*p* ≤ .05; *r* = .15). ILS total score and subscale scores showed no correlation with workload scores.

Path analysis (SEM) to test predictive validity showed that the ILS total and subscale scores are significantly associated with ORIC total and subscale scores. In the bivariate path models, the influence of knowledgeable leadership on organizational change efficacy is the only one that was not significant, whereas perseverant leadership showed the strongest associations with ORIC (see Table 3).

**Table 3:** Bivariate and full model of Implementation Leadership Scale (ILS) scores, subscale scores and organizational readiness for implementing change (ORIC) scores and subscale scores

	Change commitment (ORIC subscale; time 2)					Change efficacy (ORIC subscale; time 2)				
	Standardized Path coefficient (S.E.)	SRMR	RMSEA	CFI	TLI	Standardized Path coefficient (S.E.)	SRMR	RMSEA	CFI	TLI
Pro-active leadership (time 1)	403^**^ (.08)	.047	.091	.975	.963	398^**^ (.08)	.059	.074	.981	.969
Knowledgeable leadership (time 1)	191* (.08)	.049	.105	.973	.960	161 (.08)	.033	.011	1.00	.999
Supportive leadership (time 1)	347^**^ (.08)	.048	.128	.959	.939	486^**^ (.07)	.023	.062	1.00	1.00
Perseverant leadership (time 1)	518^**^ (.07)	.021	.084	.980	.971	506^**^ (.07)	.029	.058	.989	.983
ILS total (time 1)	545^**^ (.08)	.065	.058	.966	.960	629^**^ (.07)	.065	.050	.972	.967

Note: *n* = 135; ILS total = second order model; ILS total (time 1) - > ORIC total (time 2) = .593** (.06), standardized root mean quare residual = .065, root mean square error of approximation =.055, comparative fit index = .968, Tucker-Lewis index = .960, *p <.05, **p <.001

## Discussion

Implementation leadership behavior, as part of the organizational culture and contextual factors that determine implementation outcomes, is important to consider for the successful implementation of EBPs in healthcare organizations. The study of the interrelationships of inherent constructs of this implementation-related research subject requires validated scales in the context of ORIC. The main result of this validation study is that implementation leadership behavior is also empirically a relevant influencing factor determining at the organization-related level. The verified psychometric properties prove that the scale can be used in primary care settings. In addition, as suggested in other studies, we examined the influence of the ILS on multiple organizational factors [23]. To the best of our knowledge, our study is the first to validate and apply the scale to a primary health care context.

Our findings confirmed the four-dimensional structure of ILS, and they are consistent with similar ILS validation studies [26–28]; however, the second-order model could not be estimated in the first measurement model. To account for the theoretically assumed relationships between ILS subscales, we allowed for covariances between subscales. Furthermore, the global fit indices as well as the local fit indices underlined the four-dimensional structure. On the basis of the good results of the measurement model, we retested the original second-order ILS model using SEM in the presence of the ORIC scale. On the basis of these calculations, the second-order ILS could be verified and adopted owing to good global and local fit indices (CFI = .968, RMSEA = .05).

Descriptive findings indicate medium agreement values for the ILS’s single items and aggregated mean values with a slight tendency toward agreement; this was especially true regarding the knowledgeable leadership dimension. A possible explanation is that the practice owners, as drivers of implementation, are the first actors in the organization to come into contact with the intervention and have also received training in the use of the software. This then leads to a higher rating of their self-assessed knowledge of the intervention and knowledgeable leadership item. Other forms of leadership behavior may not have received as much training or education among PCPs in private practice, and they may not perceive themselves to be in a managerial role in their practice. Such self-perception is indicated by proactive leadership behavior scores, which measure behavior related to managing activities during the implementation process and were below the middle agreement category for both single items and on average.

The analysis of convergent validity provides insights into the relationship of the ILS with other constructs theoretically related to the conceptualization of the inner setting in CFIR. All IL subscale scores showed moderate correlation with organizational innovation climate as an organizational context factor. Although organizational climate has been used in other studies to validate the ILS for discriminant validity, our study showed significant correlations between the constructs [18]. This finding may be related to the fact that the constructs used in other validation studies have been conceptualized differently in comparison with our study, and related items focus on other aspects of organizational climate. In addition, a different conceptual background was chosen for the selection of the constructs in our study by integrating the inner setting description of CFIR. However, a study by Hower et al. (2019) examining the relationship between leadership behavior and innovation climate, as part of the organizational climate construct, confirms our content interpretation and also chose a construct of innovation climate similar to that used in our study [39]. Although the ILS total score and social capital scores also showed a significant correlation, it was only small, as all other ILS subscales showed no correlations with the social capital scores. The significant correlation of the supportive leadership subscale scores with the social capital scale scores was slightly higher than that of the ILS total score and plausible in terms of content because both scales measure aspects of social support within the organization. To select constructs unrelated to the ILS to demonstrate discriminant validity, we used the single item of PCP workload. Although physicians’ perceived workload may have been higher owing to additional tasks related to the implementation, our data indicated that PCPs’ perceived workload did not affect leadership behavior, and no significant correlations were found.

Criterion-related data analysis showed the predictive validity of leadership for ORIC. The criterion-related analytical approach used in our study was rigorous regarding the temporal element, as the ILS measurement data were collected prior to the data collection of the ORIC. The PCPs’ leadership behavior showed medium to high associations with organizational change commitment and change efficacy. This is consistent with other empirical findings suggesting that leading persons, as change agents, affect team members’ willingness to change through their own change behavior [43]. The strongest predictive relationship has been shown between perseverant leadership behavior and commitment to organizational change. One possible explanation is that implementation processes are almost always accompanied by barriers that can only be overcome through perseverant leadership behavior. This behavior in turn has a strong positive influence on organizational commitment and may act as a facilitator in the implementation process. A comparison between the two surveys showed that leadership behavior did not influence the ORIC as strongly over time although the mean values of ORIC remained stable between time points. Over time, other factors may have also influenced the ORIC, for example, seasonal events such as flu outbreaks or the waves of the COVID-19 pandemic, unmet expectations for the project itself, or changes in leadership behavior during implementation.

In the context of the influence of knowledgeable leadership behavior, it is interesting to observe this variable in relation to organizational measurement tools. In the descriptive evaluations, this leadership behavior was rated the highest, whereas the path coefficients of organizational change commitment and organizational change efficacy were only very weakly or not at all associated with the knowledgeable leadership type. One possible explanation for this phenomenon is that the knowledge dimension does not play a relevant role in the social interactions of the organization related to change processes; it may have a considerably greater significance for an individual leader. Another would be that the knowledge dimension may have had a prominent importance in relation to the phase shortly after the decision of adopting the innovation. However, the findings indicate that the knowledge dimension has little to no significance in convincing the PCPs’ team members to adopt the intervention. Social skills reflected in supportive leadership behaviors appear to have a stronger impact on organizational team members as personal appreciation for their work is expressed. This seems to be a common paradox in knowledge translation processes and EBP implementation: On the one hand, participants need specific knowledge to apply a new practice. On the other hand, the social processes of the organizational context play an equally important role in acquiring the knowledge in the first place and successfully adopting it into practice. The individual decision to adopt the innovation, in line with leadership behavior, may have had a positive influence on ORIC at the beginning of the intervention [33, 34]. Further research is needed to investigate whether, for example, centralization of decision making by PCPs in the initiation phase may have a positive effect on ORIC and why the influence changes during the implementation process (time 2).

### Strengths and limitations

The results presented must be interpreted in light of the methodological limitations. The high path coefficient between ILS and ORIC may indicate that the constructs conceptually overlap although the two instruments assess at different levels of analyses (micro and meso levels, respectively). Additional assessment has shown that the indicators of all subscales of ILS and ORIC are positively correlated with their associated constructs and explain over 50% of the variance in the indicators (AVE). Only two of the IL subscales (proactive leadership and perseverant leadership behavior) and their indicators share variance with ORIC. Discriminant validity is slightly violated in these relationships. For calculating discriminant validity, we used a strict criterion in these analyses; discriminant validity was assumed only if all AVE values are greater than all squared correlations of latent variables with any other latent constructs. With regard to the discriminant validity of the individual subtypes of ILS, good results were obtained in accordance with the threshold values. Recently presented methodological approaches recommend further analyses to investigate discriminant validity [44]. In addition, some of the bivariate path models used to calculate predictive validity showed RMSEA values above the threshold values. We assume that this is owing to the small number of degrees of freedom in these models [45]. The RMSEA values in the full model and in the overview with the other indices have shown good model fit. Furthermore, another limitation of our study that needs to be discussed is the use of self-assessment instruments. These instruments may bias the results, as self-assessment of one’s own abilities and behaviors lead to both underestimation and overestimation. There are several methodological approaches to address problems in self-assessment of leader behavior in general (e.g., by examining convergence [i.e., correlation] between leader and observer ratings) or specifically for physicians’ self-assessment abilities [46–49]. From a research practice perspective, it is also important to clarify who the appropriate observers are for the behavior being measured and whether access to them is possible. Incentives to participate in our primary formative evaluation study were offered only to participating physicians, as the intervention primarily involved a physician activity: prescribing medications. We did not include a separate research question about the perspective of practice staff regarding the intervention although our primary data had indicated that physicians involved their staff and delegated tasks related to the use of the software. Therefore, we did not have the possibility to compare the self-assessment with another (external) assessment in our secondary analysis. Despite the limitations that may occur when using self-assessment measurement instruments, data analyses provided us with important information about areas for improvement in the implementation of the intervention and how they are rated by physicians as the main users of the intervention in practice and the main actors in its implementation. In this sense, their self-assessment is of particular importance for our research question [50]. Furthermore, our findings may be specific for primary care settings and should be tested in other settings in Germany to extend the evidence base of a valid and reliable ILS.

### Implications for research and practice

Researchers or organizations may apply the present findings to identify areas for improvement in implementation leadership in their healthcare organizations. Particularly in primary care, further research is needed to examine the effectiveness of implementation strategies focusing on leadership development for PCPs. In addition, the study results suggest that it may also be necessary to investigate how and why the distinct types of leadership behaviors have different effects related to the time point in the implementation process. Some evaluation studies have already considered the temporal element [51]. The organizational approach underlying ILS and management theory (especially with regard to the concept of absorptive capacity), as illustrated, views leadership in the change process as a concept oriented toward strategic capabilities [17, 23]. In contrast to the theory, our empirical results suggest that leadership behavior cannot be interpreted exclusively as a strategic capability. Professional self-concepts may also influence self-assessment of leadership behavior, for instance, the salient low ratings of the proactive leadership behavior type or the high ratings of the knowledgeable leadership type [13]. These findings highlight the need to examine the various occupational group-specific patterns of leadership behavior types in healthcare organizations. As the empirical analyses have shown, the distinct types of leadership behaviors have different effects on the investigated organization-related factors. Assuming that the behavior types are also associated with certain skills, it would also be relevant to clarify which skills are particularly important during the implementation process in practice.

## Conclusion

The ILS is a brief instrument that can be used in health services research to investigate the effects of leadership behaviors during change processes in healthcare organizations and to evaluate interventions to promote supportive implementation activities by key personnel; however, its sensitivity to temporal elements has not been fully demonstrated. In particular, the confirmed associations of the ILS or its subscales with social capital, innovation climate, and ORIC point to the relevance of implementation leadership behavior as a significant resource in the implementation process of innovations in healthcare organizations.

## Data Availability

The datasets generated and analyzed in the current study is not publicly available due to participant consent restricting data use to the research team. With permission of the AdAM consortium partners, represented by the Head of Project Management (BARMER, Wuppertal, Germany) (Petra.Kellermann-Muehlhoff@barmer.de), the data can be made available upon reasonable request.
